# A study on the impact of high-speed rail on the consumption structure of urban and rural residents in China

**DOI:** 10.1371/journal.pone.0307947

**Published:** 2024-10-25

**Authors:** Yafei Xu, Guoli Ou

**Affiliations:** School of Economics and Management, Beijing Jiaotong University, Beijing, China; National University of Sciences and Technology, PAKISTAN

## Abstract

Consumption is crucial to individual well-being and national economic development. This study investigates whether high-speed rail (HSR) influences consumption expenditure (CE) and consumption structure (CS) of urban and rural Chinese citizens. Using panel data from 2003 to 2019 and econometric models, this study finds that: (1) HSR significantly increases CE for both urban and rural residents, promotes CS upgrades in rural areas, but inhibits CS upgrades in urban areas. These results remain robust after extensive testing. (2) HSR’s impact on urban consumption is relatively focused and singular, whereas its effect on rural consumption is dispersed and extensive. Additionally, the impact of HSR on consumption exhibited significant delays and regional characteristics. (3) Mediation analysis reveals that HSR significantly enhances urban and rural CE and facilitates rural CS upgrades through market, price, and income effects. However, it also triggers housing price increases, impeding urban CS upgrades. This study provides important references for the government to optimize transportation infrastructure investments, promote balanced economic development between urban and rural areas, and enhance residents’ well-being.

## 1. Introduction

High-speed rail (HSR) is undoubtedly one of the most significant breakthroughs in rail transport technology development in the second half of the 20th century [1]. Since the world’s first HSR line, the Japanese Shinkansen, began operations sixty years ago, HSR has rapidly expanded to all developed countries [2]. Academic study has focused on the link between transportation infrastructure and economic growth since classical economic growth theory highlighted that infrastructure, such as transport, is necessary for economic growth [3–7]. With the rapid development of HSR, the connection between HSR and economic development has also garnered increasing attention [8–13]. Numerous studies have shown that HSR not only significantly reduces geographical distances and travel times but also positively impacts regional economic development and industrial distribution by promoting the efficient flow of resources. Jiao et al. found that the construction of HSR in China positively contributes to economic growth, becoming a crucial force in reshaping China’s spatial economic landscape [14]. Wang et al. (2019) discovered that HSR significantly facilitates short-term population mobility, promotes industrial upgrading, and improves the quality of urbanization [15]. However, research on the relationship between HSR and consumption is relatively sparse. Although some scholars have explored the impact of HSR on consumption from specific angles such as tourism [16–18], financial transactions [19], and housing prices [20], whether HSR promotes the upgrading of consumption structures and the underlying mechanisms remain to be thoroughly explored.

**Fig 1:**
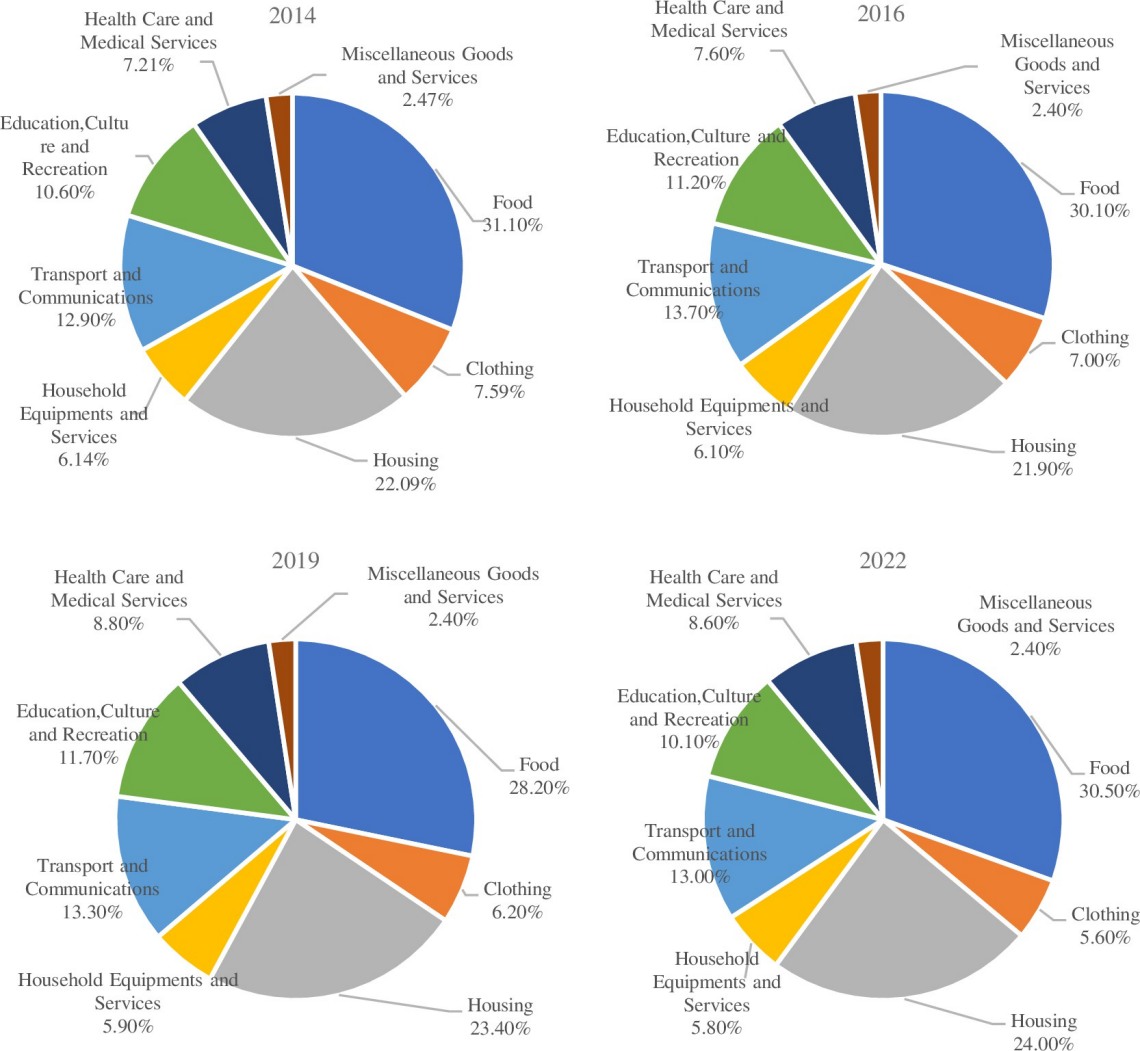
Consumption structure of Chinese residents in 2014, 2016,2019, and 2022.

Consumption, as a core driver of national economic growth, not only directly affects people’s quality of life but also plays a crucial role in shaping the country’s economic growth model and driving momentum transformation [21, 22]. According to authoritative data released by the National Bureau of Statistics of China, the contribution rate of consumption to economic growth in China reached 82.5% in 2023. This figure fully demonstrates the importance of consumption in the national economy. Amidst escalating international trade frictions and the dual challenges of high-end consumption outflow and low-end commodity overcapacity in China’s consumer market, effectively tapping into the consumption potential of domestic residents and optimizing the consumption structure to promote healthy internal economic circulation have become key research issues. By comparing data on the composition of Chinese residents’ consumption expenditures in recent years (Fig 1), we find that, excluding the impact of the COVID-19 pandemic, the proportion of survival-oriented consumption represented by clothing and food has gradually decreased, while the focus of residents’ consumption is shifting towards development-oriented and enjoyment-oriented consumption. However, the proportion of housing expenditures has shown an upward trend. In the face of shifting and evolving consumption patterns, what impact does HSR have on the changes in residents’ consumption structures? What are the underlying mechanisms? Furthermore, what intrinsic connections exist among HSR development, housing price fluctuations, and the upgrading of consumption structures?

Unfortunately, the academic field has yet to provide clear answers to these questions. Additionally, our further investigation revealed the following research gaps: First, within the context of a dual economic structure, there is a lack of studies that analyze the economic effects of HSR from both urban and rural perspectives. Second, considering the phased nature of HSR construction, existing research often fails to fully capture the dynamic effects of HSR and provides insufficient exploration of the influencing mechanisms. Based on this, our study adopts the urban and rural dimensions as the research perspective. Utilizing panel data from 30 Chinese provinces from 2003 to 2019, we employ a difference-in-differences (DID) model and a mediation effects model to empirically analyze the impact of HSR on the consumption expenditure and consumption structure of urban and rural residents, as well as its underlying mechanisms.

The study reveals that (1) HSR significantly drive the growth of urban and rural residents’ CE, particularly showcasing a notable role in promoting the upgrading of CS among rural residents, while exerting a certain inhibitory effect on the upgrading of CS among urban residents. This conclusion holds firm even after undergoing a series of robustness tests, including parallel trend tests, replacement of explanatory variables, propensity score matching difference-in-differences (PSM-DID) method, and instrumental variable method. (2) In the analysis of heterogeneity, we delve into the impact of HSR on different types of consumption, various stages of development, and different regions. The results indicate that the influence of HSR on urban residents’ CE primarily concentrates on housing and education-cultural entertainment items, while it demonstrates multifaceted promotion effects on rural residents’ CE, such as in education-cultural entertainment, transportation-communication, health care and medical services, clothing and housing sectors. Additionally, the impact of HSR on consumption exhibits a lag effect, notably promoting urban and rural residents’ CE after 2008 and significantly driving the upgrading of CS among rural residents after 2013 while restraining the upgrading of CS among urban residents. Simultaneously, we also uncover significant regional characteristics in the impact of HSR, notably stimulating CE and CS upgrading in eastern rural areas, yet exerting an inhibitory effect on the CS upgrading of urban residents in central and western regions. (3) The mediation analysis unveils that HSR significantly propel the CE of urban and rural residents and upgrading of CS among rural residents through market effects, price effects and income effects. Meanwhile, HSR stimulate housing price increases, thereby restraining the upgrading of CS among urban residents.

The marginal contribution of this paper is primarily manifested in the following aspects: Firstly, departing from both urban and rural dimensions, it empirically analyzes the impact of HSR on CE and CS, thereby filling the research gap in the field of HSR and consumption, and providing the academic community with a more comprehensive and in-depth perspective. Secondly, by introducing multiple intermediate variables such as income, price, marketization, and housing prices, it comprehensively and systematically explores the mediating effects of HSR on the consumption process, deepening the understanding of the economic impact mechanisms of HSR. Thirdly, through further refining the classification of consumption types, development stages, and regional positions, it reveals the differentiated characteristics of HSR impact on consumption, providing policymakers with powerful references and insights on how to promote consumption, enhance residents’ well-being, narrow the urban-rural development gap, and facilitate regional coordinated development through optimizing the layout of transportation infrastructure.

The essay is structured as follows: Section 2 shows the research context and theoretical analysis. along with the research hypotheses. Section 3 presents the model and data used in the study, followed by the empirical results in Section 4. Finally, Section 5 gives the conclusions and discussion.

## 2. Research context and theoretical analysis

### 2.1 Classification of the consumption structure

**Fig 2:**
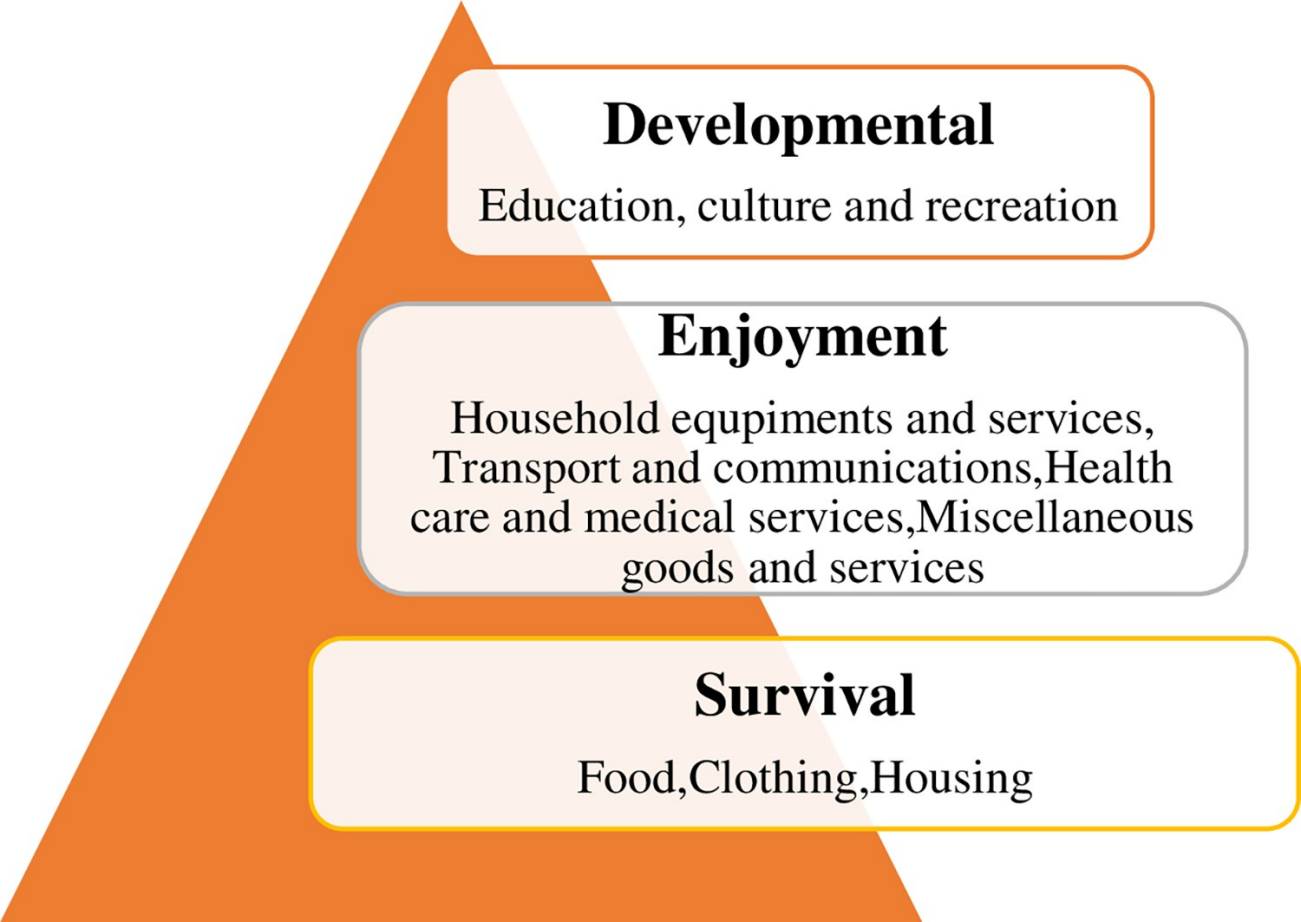
Classification of consumption structure.

According to the Classification of Consumer Expenditure 2013 released by the National Bureau of Statistics, CE is divided into 8 main categories as follows: Food, Clothing, Housing, Household equipment and services, Transport and communications, Education, culture and recreation, Health care and medical services, Miscellaneous goods and services. Based on the work of Taylor and Houthakker [23]and Aschauer et al. [24], CE can be further divided into survival consumption, enjoyment consumption and developmental consumption according to different levels of consumption needs. In this paper, as the share of enjoyment and developmental consumption in total consumption increases, it indicates that residents are seeking a higher level of consumption and that CS is gradually being optimized and upgraded (Fig 2).

### 2.2 Theoretical analysis

**Fig 3:**
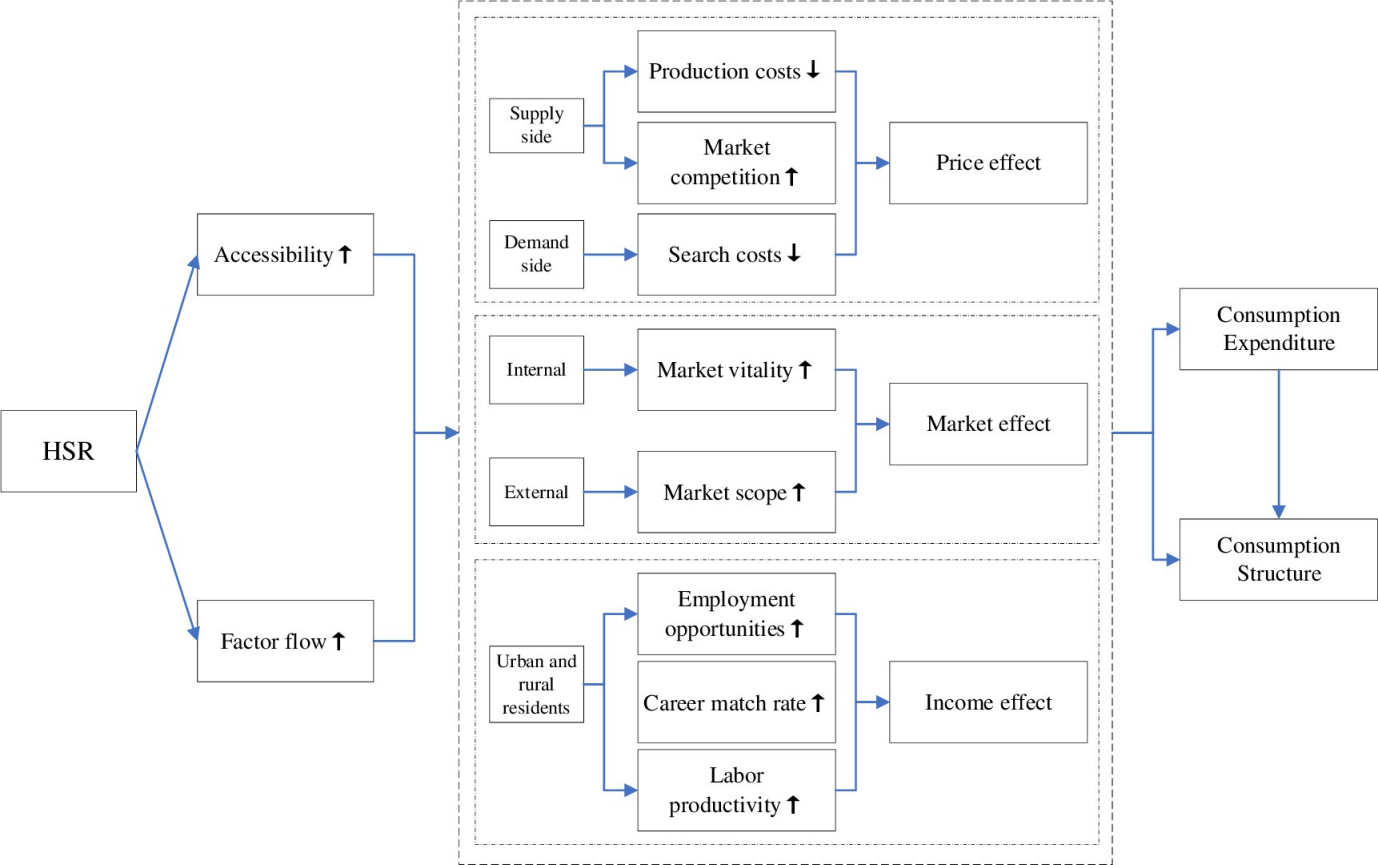
The structure of theoretical analysis 1.

This paper argues that HSR will stimulate the consumption of urban and rural residents through the price effect, market effect and income effect, leading to an increase in CE accompanied by a restructuring of consumption (Fig 3). The specific analysis is as follows:

First, the price effect refers to how HSR affects the price of goods or services, influenced by factors such as production costs, market competition and search costs. From a supply-side perspective, HSR improves accessibility between regions [14, 25, 26], leading to savings in transportation costs [5, 27]. It also enhances the efficiency of production materials flow and allocation, fostering industrial agglomeration [28, 29], thereby reducing local production costs. Moreover, by increasing the scale and speed of goods movement, HSR reduces information constraints caused by imperfect information and stimulates market competition, resulting in lower commodity prices [30, 31]. From a demand-side perspective, HSR effectively reduces consumers’ search costs. In addition to accelerates the logistics and information flow, HSR breaks the time and space constraints, expands the scope of consumers’ search, increases searches frequency and improves the search efficiency, enables consumers to find their own satisfactory products in a short time, and reduces the unit search cost [13, 32].

Second, the market effect refers to the HSR’s effects on the consumer market by changing the dynamics and size of the market. The changes in market dynamics brought about by the HSR are mainly reflected in the enhanced entry of external market players and capital inflows due to lower transportation costs and inventory cost savings [33]. External enterprises, with the help of convenient local transport infrastructure, enhance the exploitation of local resources and promote local industries, resulting in better development of the local market, improved market dynamics and enrichment of the quantity and variety of consumer goods in the local market. In addition, HSR has led to changes in market size, mainly by improving connectivity between cities, increasing accessibility and availability, reducing transaction frictions, expanding the range of consumer markets for residents [34], and providing more consumer choices in external markets.

Third, the income effect refers to the consequence of HSR on changes in economic income and income levels of residents through the creation of more jobs, higher occupational matching rates and higher labor productivity. And the level of wealth directly influences the CE level and the proportion of consumption of different types of products or services by urban and rural residents [35]. The investment and construction of HSR will not only directly increase employment, but also promote the development of new sectors, industries and business models, which will indirectly create more jobs [10, 36], optimize the employment environment and increase the regional employment rate [37]. HSR has enhanced the accessibility of employment information, reduced the cost of job search, reduced the pressure of labor mismatch and improved the efficiency of matching career choices through accelerating the movement of individuals and information [38]. Moreover, the opening of HSR promotes face-to-face exchanges [39, 40] and the diffusion of tacit knowledge [41], effectively promoting the concentration of human capital and knowledge elements in cities [38], while enhancing the vitality of urban scientific and technological innovation [42–45], which is favorable to improving the level of talent and increasing the return on human capital [34].

However, China’s economic development exhibits a prominent dichotomous structure, with urban areas significantly outpacing rural areas in development due to their well-established infrastructure and advanced economic status. Simultaneously, disparities exist between urban and rural residents in education levels, income levels, age structure, and consumption attitudes, introducing differentiation in the impact of HSR on the CS of urban and rural areas. Therefore, based on the previous analysis, the research hypotheses of this paper are proposed:

**H**_**1**_ **: HSR facilitates an increase in CE among urban and rural residents.**
**H**_**2**_ **: HSR promotes the CE of urban and rural residents through the price effect, market effect and income effect, and the impact on the CS of urban and rural residents exhibits differentiation.**

**Fig 4:**
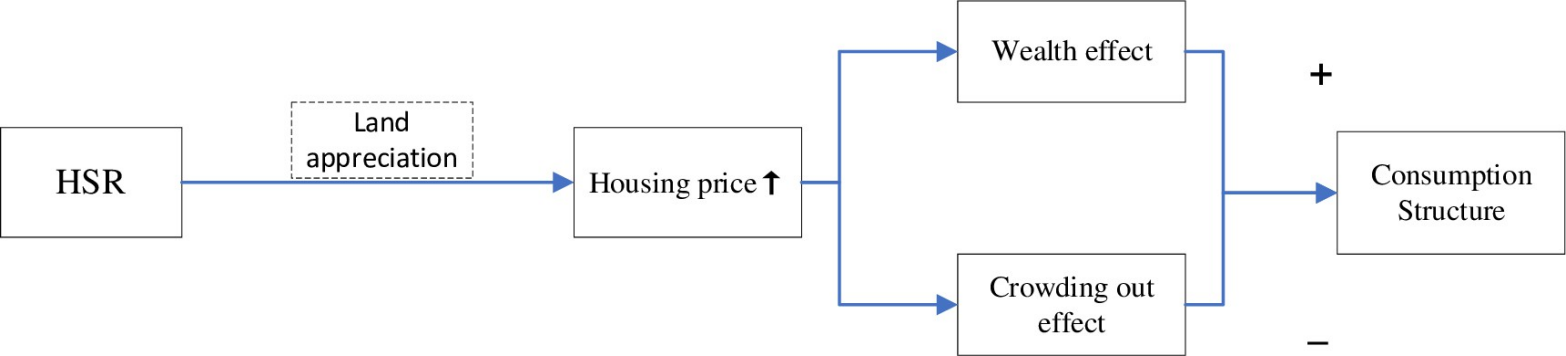
The structure of theoretical analysis 2.

Housing cost is an essential part of residents’ consumption and affects the upgrading of their CS. On the one hand, rising housing prices raise the value of homeowners’ household wealth, creating a wealth effect [46]. With the increase in wealth, residents’ consumption will tend to develop enjoyment-oriented consumption such as education, medical care and entertainment, thus promoting the upgrading of residents’ CS. On the other hand, the boost in housing prices will lead to an increase in residents’ living costs, which will crowd out their developmental or enjoyment-oriented consumption, creating a crowding out effect that is not conducive to the upgrading of CS. According to land rent theory, increased accessibility to goods and services can contribute to local land appreciation [47]. The HSR has increased accessibility between regions and convenience for residents, while pushing up property prices in surrounding areas along the lone [20, 48]. Property is not only an investment, but also a consumer good. For some residents, home equity is an important part of their wealth, and the increase in the value of home equity will undoubtedly increase the total household wealth, stimulating residents’ willingness to spend on development and enjoyment-oriented consumption, thereby promoting the upgrading of their CS in a progressive direction. It also means that another part of the population will have to reduce their current consumption to buy or rent a house, which is not conducive to the upgrading of the CS (Fig 4). Base on the previous analysis, the last research hypothesis is proposed:

**H**_**3**_ **: HSR has a dual effect of the “wealth effect” and the “crowding out effect” through increasing house prices, resulting in uncertainty regarding its impact on the CS of urban and rural residents.**

## 3. Model and data

### 3.1 Model specification

#### 3.1.1 Difference-in-differences model (DID)

In this part, we examine the consequence of HSR on the CE and CS of urban and rural residents in China by establishing the following continuous DID model based on the research of Shao et al. [49], Lin [37], Nunn and Qian [50]. The model can be expressed as follows:

$$Y_{it}=\partial_{0}+\partial_{1}{HSR}_{it}+{\partial_{2}control}_{it}+\mu_{i}+\gamma_{t}+\varepsilon_{it}$$ (1)

Where *Y* is CE or CS. *HSR* represents number of HSR lines. ∂_1_ is the net impact of HSR on the CE or CS, which is the key coefficient in this study. *control* is a set of control factors. *μ* and *γ* represent the city fixed effect and the time fixed effect, respectively. *ε* means the error term, *i* is the province, *t* is the year.

#### 3.1.2 Mediator effect model

A recursive regression equation is used to explore what is the mechanism of HSR’s effect on the urban and rural residents’ CE and CS. Following the study of Hayes et al. [51], we add the following two models based on model (1):

$$M_{it}=\alpha_{0}+\alpha_{1}{HSR}_{it}+\alpha_{2}{control}_{it}+\mu_{i}+\gamma_{t}+\varepsilon_{it}$$ (2)

$$Y_{it}=\beta_{0}+\beta_{1}{HSR}_{it}+\beta_{2}M_{it}+\beta_{3}{control}_{it}+\mu_{i}+\gamma_{t}+\varepsilon_{it}$$ (3)

Where *M* is the mediator variable, the remaining variables are the same as in model (1). If *α*_1_, *β*_1_ and *β*_2_ are all significant, and *β*_1_<∂_1_, the partial mediation effect exists.

### 3.2 Variable specification

#### 3.2.1 Dependent variable

The dependent variables are the consumption expenditure (CE) and consumption structure (CS) of urban and rural residents. First, CE is calculated as the logarithm of per capita CE of urban and rural residents, and its real value is taken into account through the use of consumer price index of each province as the base period in 2003. Second, CS is determined by the proportion of consumption allocated to development and enjoyment in total consumption. A higher value of CS indicates a more advanced CS among the region’s residents.

#### 3.2.2 Independent variable

High-speed rail (HSR). It is measured by the number of HSR lines opened in each provincial administrative region. The data for this indicator were manually collected and compiled by the author, primarily based on the "China Railway Passenger Train Schedule" and the 12306 China Railway website. These sources detail the departure and terminal stations of each train, as well as the names of the stops along the route. To construct the core explanatory variable, we specifically selected information from high-speed trains marked with the prefix "G." For instance, if high-speed rail line G1 opened in 2003 and passed through provinces A and B, we assigned a value of 1 to the HSR indicator for both provinces A and B in the 2003 data. Similarly, if high-speed rail line G2 opened in 2004 and passed through provinces A, B, and C, we assigned a value of 2 to the HSR indicator for provinces A and B, and a value of 1 for province C in the 2004 data. This process was repeated and accumulated accordingly.

#### 3.2.3 Control variables

①Economic development (PGDP): GDP per capita for each province is selected to evaluate the level of localized economic growth. It is used to control for the impact of economic development on consumption. ②Financial development (FIN): Calculated as the ratio of financial institutions’ deposits and loans to GDP. Referring to the study by Li et al. [52], this variable is used to control for the impact of financial development on household consumption. ③Government expenditure(GOV): Measured by provincial fiscal spending as a share of GDP. It is used to control for the impact of government intervention on residents’ consumption choices. ④Foreign direct investment (FDI): Calculated as the proportion of real FDI to GDP, with real FDI converted into RMB using the average exchange rate of the year. Cities with higher levels of openness can effectively attract foreign investment, enrich the variety of local products, and stimulate consumer spending.⑤Industrial structure (STR): Expressed as the proportion of value added in the tertiary sector to that in the secondary sector. The adjustment and optimization of industrial structure will impact supply and demand, as well as change consumption habits and preferences, thereby affecting the structure of residents’ consumption.⑥House price (HP): Determined by the average sales price of residential commercial properties, chosen as a proxy for house prices. Housing expenditure is a key component of Chinese residents’ consumption expenditure. This indicator is used to control for the potential crowding-out effect of housing expenditure on other types of consumption.

### 3.3 Data

**Fig 5:**
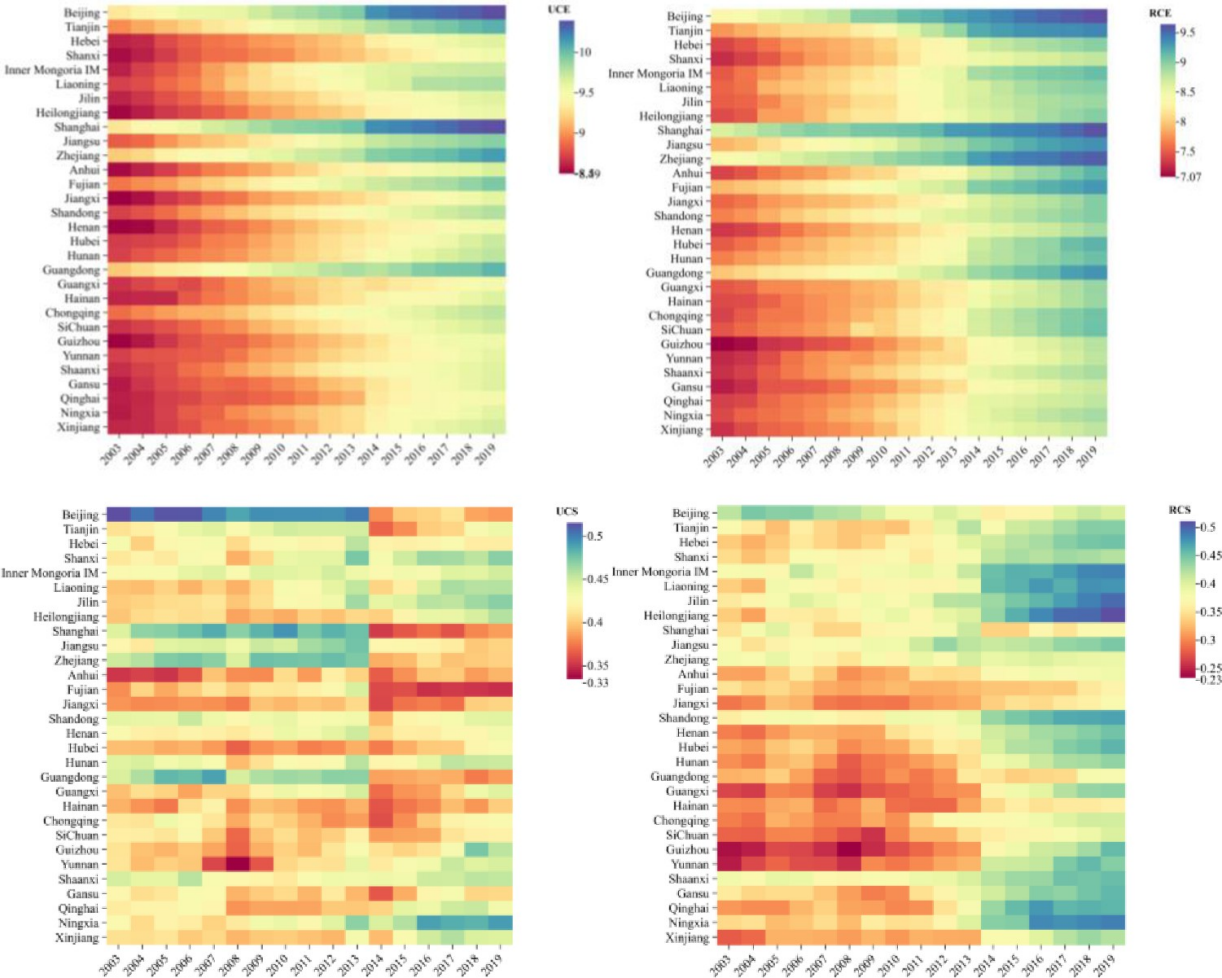
The changing trend of CE and CS in urban (UCE, UCS) and rural (RCE, RCS) areas.

**Fig 6:**
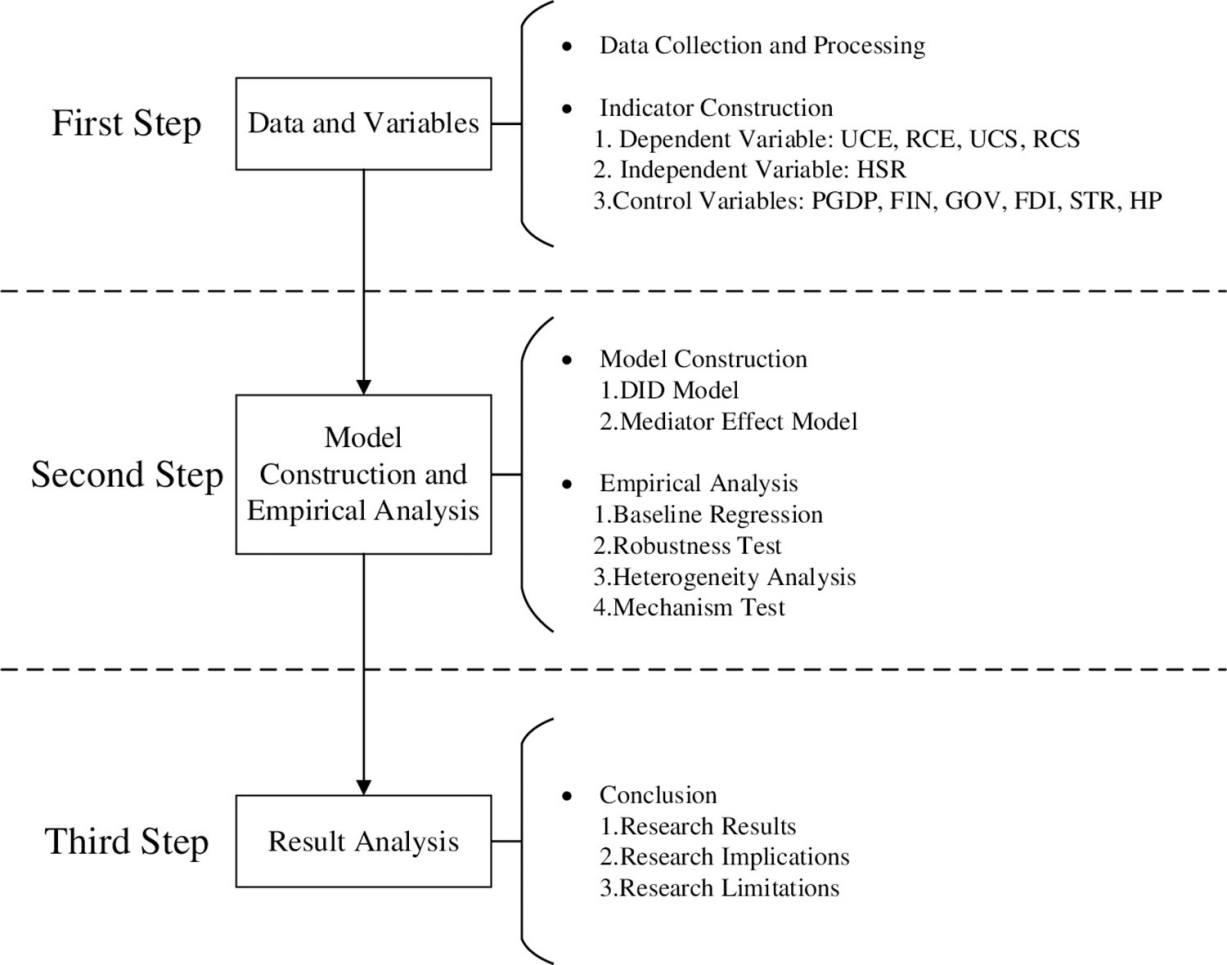
Research steps.

The research sample selected data from 30 provincial administrative regions nationwide from 2003 to 2019, excluding Macau, Tibet, Hong Kong, and Taiwan due to data availability. Data were obtained from the website of the State Railway Corporation,12306, EPS, China City Statistical Yearbook and China Statistical Yearbook. For detailed statistics, see Table 1 and Fig 5. Additionally, the research steps are shown in Fig 6.

**Table 1:** Descriptive statistic.

	Variables	Symbols	N	Mean	Sd	Min	Max
Dependent Variable	Urban consumption expenditure	UCE	510	9.282	0.405	8.500	10.390
	Rural consumption expenditure	RCE	510	8.343	0.577	7.078	9.644
	Urban consumption structure	UCS	510	0.420	0.032	0.335	0.516
	Rural consumption structure	RCS	510	0.367	0.056	0.234	0.511
Independent Variable	High-speed rail	HSR	510	3.318	3.919	0.00	19.00
Control Variable	Economic development	PGDP	510	10.300	0.737	8.218	11.994
	Financial development	FIN	510	2.753	1.071	0.956	7.506
	Government expenditure	GOV	510	0.226	0.119	0.017	1.688
	Foreign direct investment	FDI	510	0.026	0.023	0.000	0.186
	Industrial structure	STR	510	1.038	0.571	0.500	5.169
	House price	HP	510	0.537	0.476	0.096	3.843

## 4. Estimation results

### 4.1 Baseline regression

Table 2 presents the regression results testing the effect of HSR on the CE and CS of urban and rural residents. Regrading CE, it can be seen that HSR significantly promotes the increase in urban and rural CE (Columns 1 and 2). The coefficient for UCE is 0.007, passing the 5% significance test, and the coefficient for RCE is 0.019, statistically significant at the 1%level. Moreover, the positive coefficients for PGDP, FIN and STR indicate that the development of the economic, financial and industrial structure in each province significantly contributes to the increase in residents’ CE. Notably, FDI exhibits a significant effect on RCE, possibly because HSR opens up rural consumer markets, leading to a greater variety of goods available in domestic cities and stimulating the consumption potential of rural residents. The control variable HP is excluded from the regression as housing expenditure is a form of CE. Concerning CS, the coefficient for UCS is significantly negative (-0.002 in column 3), passing the 10% significance test, suggesting that HSR has a suppressive effect on the upgrading of urban residents’ CS. However, the coefficient for RCS is 0.002 (Column 4), passing the 10% significance test, indicating that HSR can encourage the upgrading of CS among rural residents. Additionally, PGDP, STR and FIN are found to promote CS, while HP suppresses CS at the 1% significance level. It can be inferred that the housing expenditure of urban and rural residents has a crowding-out effect on the enhancement of CS, while the HSR may drive up the housing prices in areas along the route, thereby suppressing the enhancement of CS.

**Table 2:** Baseline regression results of the DID model.

	(1)	(2)	(3)	(4)
	UCE	RCE	UCS	RCS
HSR	0.007**	0.019***	-0.002*	0.002*
	(2.56)	(3.69)	(-1.76)	(1.89)
PGDP	0.431***	0.613***	0.026***	0.050***
	(24.86)	(23.06)	(4.76)	(8.67)
FDI	-0.416	1.258***	-0.075	0.162
	(-1.30)	(3.08)	(-0.78)	(0.88)
STR	0.153***	0.183***	0.020**	0.056***
	(6.47)	(3.91)	(2.14)	(4.05)
GOV	0.037	0.045	-0.009	-0.020
	(0.85)	(1.15)	(-0.55)	(-1.02)
FIN	0.067***	0.118***	0.007*	0.028***
	(5.50)	(5.29)	(1.98)	(5.41)
HP			-0.069***	-0.078***
			(-8.44)	(-7.89)
Constant	4.486***	1.416***	0.158***	-0.248***
	(26.06)	(5.52)	(3.00)	(-3.97)
Time FE	YES	YES	YES	YES
City FE	YES	YES	YES	YES
N	510	510	510	510
R^2^	0.970	0.963	0.276	0.731

Note: t statistics in parentheses.

***, **, and * = p<0.01, P<0.05, P<0.1, respectively.

### 4.2 Robustness test

#### 4.2.1 Parallel trend test

When conducting policy evaluations, the cornerstone of the DID model is to satisfy the parallel trends assumption. This requires that the treatment group and the control group follow the same developmental trajectory prior to the implementation of the policy intervention. Based on this premise, this study employs the event study method to validate the parallel trends. Specifically, we define dummy variables d_1 to d_10, representing the 1 to 10 years prior to the opening of HSR, and set current as the year when the HSR officially commenced operation. Additionally, we introduce dummy variables d1 to d10 to denote the 1 to 10 years following the HSR opening. If the parallel trends assumption holds, there should be no significant differences in the CS and CE indicators between HSR cities and non-HSR cities before the HSR opening, meaning that the coefficients of d_1 to d_10 should not be statistically significant.

**Fig 7:**
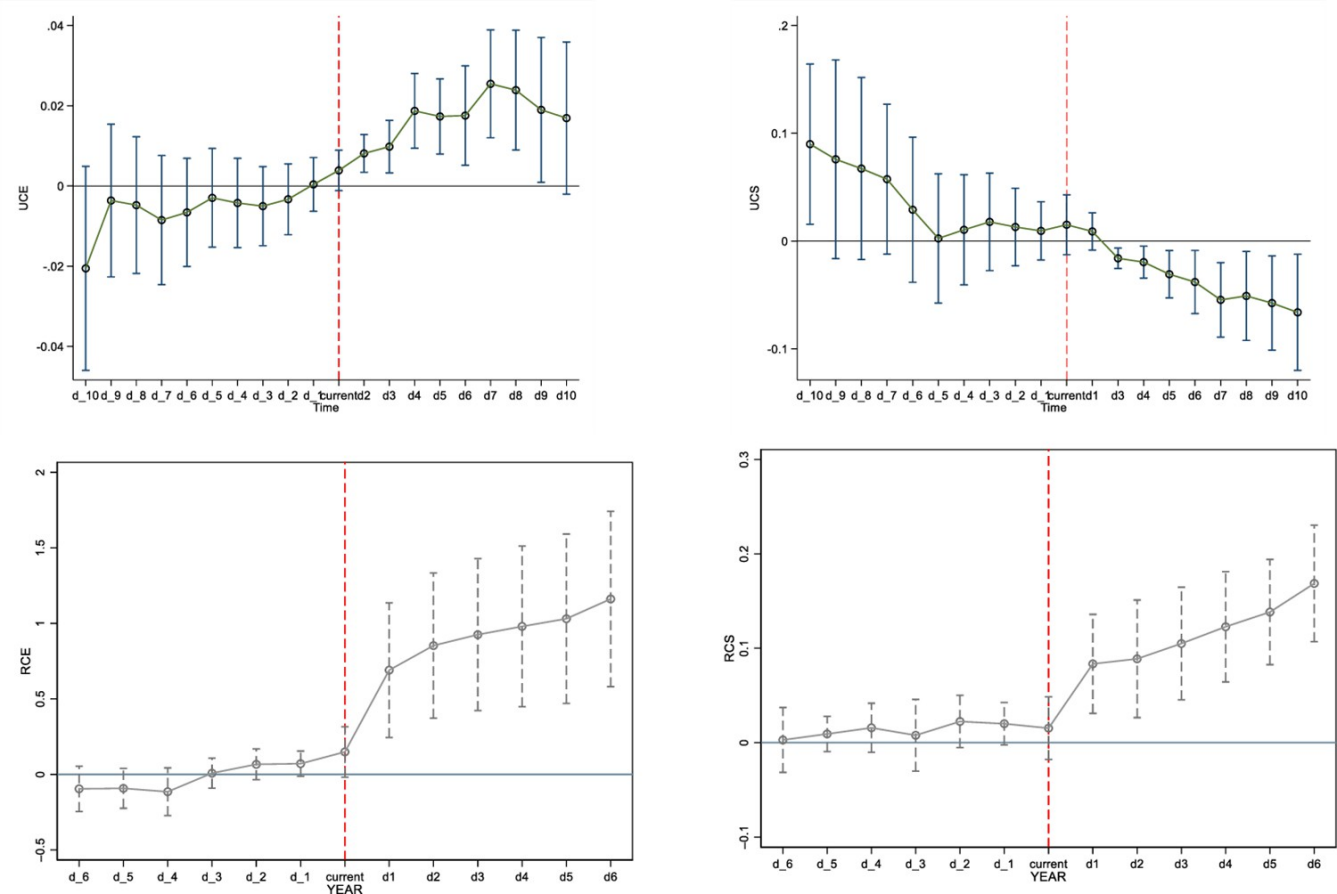
Results for parallel trend test.

As shown in Fig 7, the CS and CE indicators within the range of d_1 to d_10 are not statistically significant, strongly indicating that there were no significant differences between HSR cities and non-HSR cities in these two indicators prior to the HSR opening. Therefore, the observed changes following the HSR opening are likely driven by this major event, thereby confirming that the parallel trends assumption in this study is valid.

#### 4.2.2 Changing the independent variable method

We employed a dummy variable for the presence of HSR (hsr) and the number of HSR stops (NS) as explanatory variables, replacing the original variable representing the number of HSR lines. The hsr variable enables us to explore the impact of the presence or absence of HSR on regional consumption, while the NS variable directly indicates that higher values represent greater factor mobility in the region. The results in Table 3 indicate that although the coefficients of NS and hsr vary in magnitude, their direction and significance remain consistent with the results from the baseline regression. This further reinforces the robustness and reliability of the study’s conclusions.

**Table 3:** Results of changing the independent variable and PSM-DID.

	Change the independent variable							
Variables	UCE	RCE	UCS	RCS	UCE	RCE	UCS	RCS
	(1)	(2)	(3)	(4)	(5)	(6)	(7)	(8)
NS	0.007*	0.022**	-0.001*	0.003*				
	(1.81)	(2.62)	(-0.91)	(1.72)				
hsr					0.051*	0.415***	-0.006*	0.035***
					(1.90)	(7.41)	(-1.29)	(4.59)
Control	YES	YES	YES	YES	YES	YES	YES	YES
Time FE	YES	YES	YES	YES	YES	YES	YES	YES
City FE	YES	YES	YES	YES	YES	YES	YES	YES
N	510	510	510	510	510	510	510	510
R2	0.949	0.917	0.219	0.696	0.936	0.700	0.264	0.566
	PSM-DID							
Variables	UCE		RCE		UCS		RCS	
	(9)		(10)		(11)		(12)	
HSR	0.010***		0.026***		-0.003**		0.003**	
	(3.27)		(4.46)		(-2.69)		(2.54)	
Control	YES		YES		YES		YES	
Time FE	YES		YES		YES		YES	
City FE	YES		YES		YES		YES	
N	300		300		300		300	
R2	0.971		0.970		0.213		0.754	

Note: t statistics in parentheses.

***, **, and * = p<0.01, P<0.05, P<0.1, respectively.

#### 4.2.3 PSM-DID

**Fig 8:**
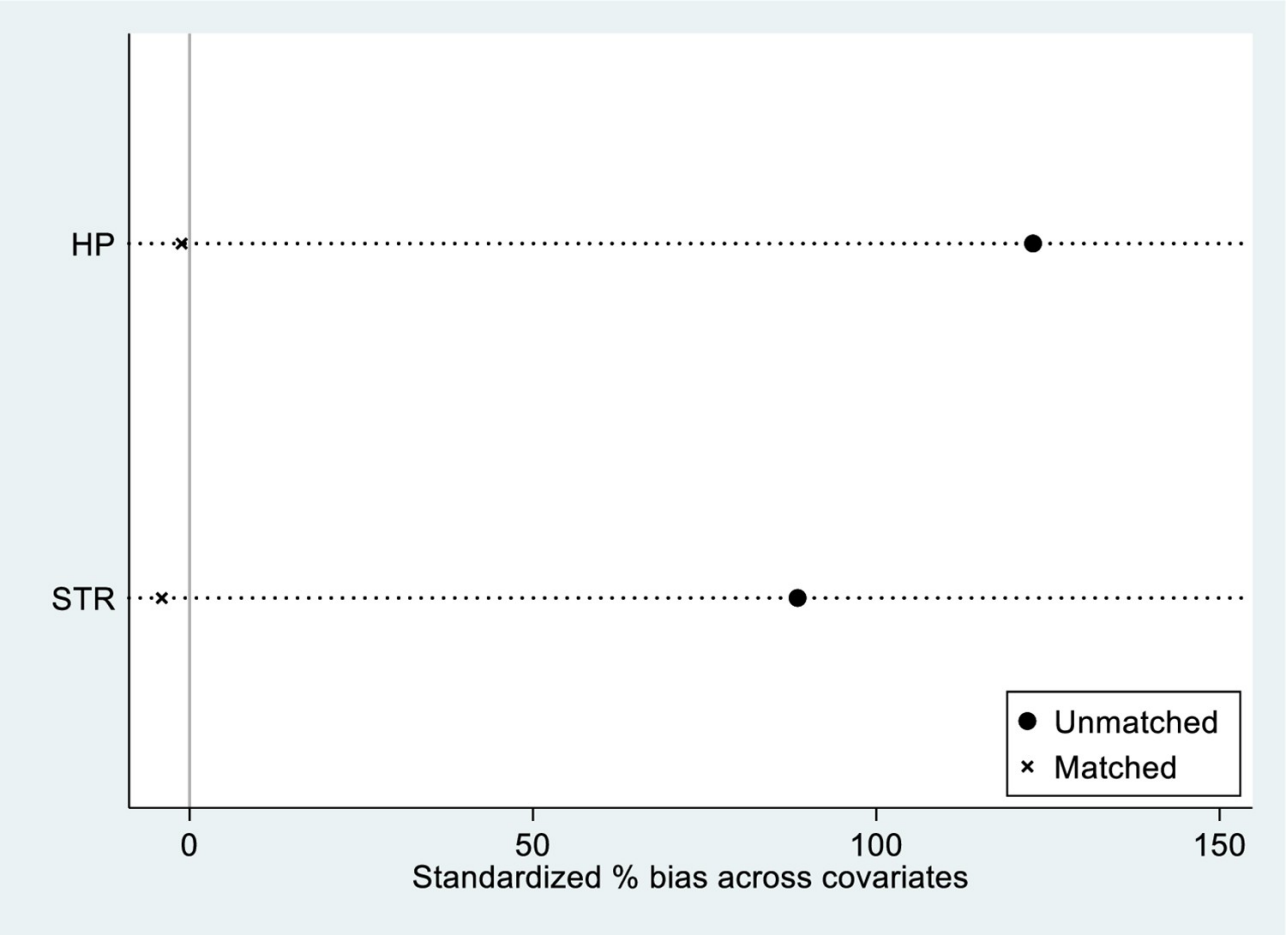
Results for balance test.

**Fig 9:**
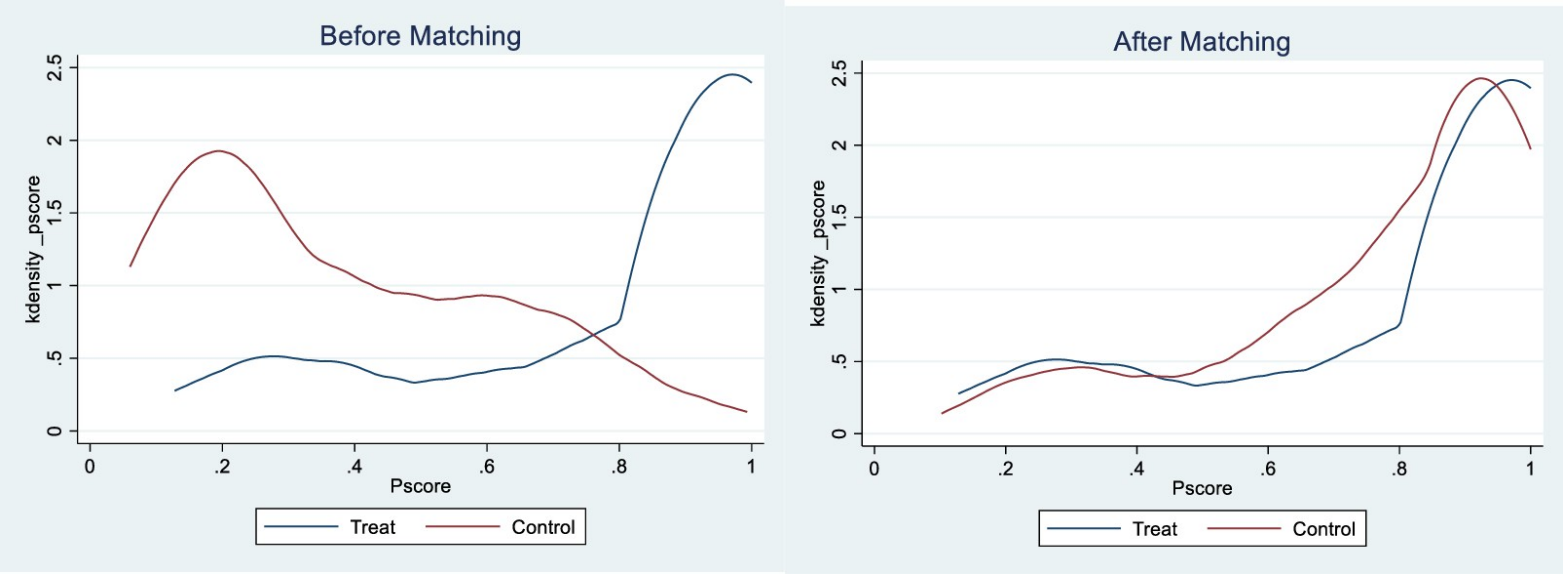
Kernel density plot.

To mitigate potential systematic differences and address issues like selection bias, this section employs PSM-DID for regression analysis. The specific steps of PSM are as follows: Industrial structure and house prices are selected as covariates, propensity scores are computed using a Logit model, and a 1:1 nearest neighbor matching is employed to pair the treatment and control groups. According to the balance test results depicted in Fig 8 the %bias of covariates is all less than 10% and significantly smaller than the absolute standard deviation before matching. Additionally, the kernel density plot in Fig 9 shows that two curves are closer after matching, indicating a significant reduction in differences between the treatment and control groups, thus validating the rationality of the matching principles and methods adopted in this section. Table 3 presents the regression results of PSM-DID. Although there are slight changes in the magnitude of the HSR coefficient, its direction and significance remain consistent with the benchmark regression results, further demonstrating the robustness of the conclusions drawn in this study.

#### 4.2.4 Instrumental variables method

By referring to the research of Duranton and Turner [53] and Zhu [54], this article uses the selection of Ming Dynasty post stations as instrumental variables (IV) to address possible endogenous problems. The reason for selecting Ming Dynasty post stations is: first, to satisfy the correlation. Due to the influence of natural factors such as geological conditions on road construction, the setting of Ming Dynasty post stations may be limited by technology, and the selection of geological conditions that are relatively conducive to construction is chosen. Modern HSR in China may further improve on the original site selection, and both satisfy a high degree of correlation. Second, to satisfy the exogenous nature. Ming Dynasty post stations were mainly built to meet military needs. According to historical records, the tasks of the postal system were mainly to deliver passengers, report military affairs, transport military supplies, etc. They were less affected by economic development levels and had experienced more than 400 years, so their impact on modern economic development levels would be even smaller. Therefore, combined with the research of this article, the setting of Ming Dynasty post stations is related to high-speed railways, but there is no correlation with modern economic development, consumption levels, consumption structure, etc., which satisfies sufficient exogenous nature. According to the results in Table 4, the P-values of the Anderson canon. corr. LM statistic is all 0.000, indicating that there is no issue of insufficient identification of instrumental variables. The Cragg-Donald Wald F statistic value is greater than 10, suggesting that the null hypothesis of weak instrumental variables is rejected, indicating that there is no issue of weak instrumental variables in the model. The estimated coefficient of high-speed rail passes the significance level test, which is basically consistent with the previous research results and verifies the robustness of the conclusions of this study.

**Table 4:** Results of IV.

2SLS	First stage	Second stage	Second stage	First stage	Second stage	Second stage
Model ID	(1)	(2)	(3)	(4)	(5)	(6)
Variables	HSR	UCE	RCE	HSR	UCS	RCS
HSR		0.008***	0.006**		-0.001*	0.004***
		(2.998)	(1.387)		(-1.801)	(3.019)
IV	0.067***			0.067***		
	(13.502)			(13.552)		
Control	YES	YES	YES	YES	YES	YES
Constant	-33.638***	4.486***	1.149***	-36.380***	0.131***	-0.444***
	(-18.244)	(40.544)	(6.321)	(-14.791)	(3.749)	(-8.084)
N	510	510	510	510	510	510
R^2^	0.562	0.939	0.919	0.564	0.319	0.448
Anderson canon. corr. LM statistic		136.61	136.61		135.66	135.66
		[0.000]	[0.000]		[0.000]	[0.000]
Cragg-Donald Wald F statistic	183.664			182.29		

Note: t statistics in parentheses.

***, **, and * = p<0.01, P<0.05, P<0.1, respectively.

### 4.3 Heterogeneity analysis

#### 4.3.1 Analysis of different types of consumption

Table 5 outlines the impact of HSR on various types of consumption expenditures among urban and rural residents. Upon conducting significance testing, we found that HSR predominantly stimulated urban residents’ spending on housing and education, culture and recreation. However, rural residents experienced a broader impact on their consumption expenditure from HSR, encompassing categories such as housing, clothing, health care and medical service, transport and communications, as well as education, culture and recreation. Notably, the promotion effect of high-speed rail on rural residents’ consumption expenditure appears to be more pronounced in comparison.

**Table 5:** Regression results for different types of consumption.

			HSR	control	Time FE	City FE	N	R^2^
Survival consumption	Food	Urban	-0.000	YES	YES	YES	510	0.906
			(-0.05)					
		Rural	0.009	YES	YES	YES	510	0.936
			(1.67)					
	Clothing	Urban	-0.008	YES	YES	YES	510	0.771
			(-1.49)					
		Rural	0.013**	YES	YES	YES	510	0.946
			(2.40)					
	Housing	Urban	0.043***	YES	YES	YES	510	0.88
			(6.85)					
		Rural	0.020**	YES	YES	YES	510	0.929
			(2.75)					
Enjoyment consumption	Household equipment and Services	Urban	0.004	YES	YES	YES	510	0.902
			(0.71)					
		Rural	0.014	YES	YES	YES	510	0.943
			(1.48)					
	Health care and Medical service	Urban	0.007	YES	YES	YES	510	0.894
			(1.58)					
		Rural	0.029***	YES	YES	YES	510	0.954
			(3.60)					
	Transport and Communications	Urban	-0.002	YES	YES	YES	510	0.917
			(-0.31)					
		Rural	0.018**	YES	YES	YES	510	0.945
			(2.61)					
	Other goods and Services	Urban	-0.005	YES	YES	YES	510	0.683
			(-0.88)					
		Rural	-0.005	YES	YES	YES	510	0.857
			(-0.54)					
Developmental consumption	Education, Culture and Recreation	Urban	0.009*	YES	YES	YES	510	0.856
			(1.87)					
		Rural	0.041***	YES	YES	YES	510	0.821
			(3.63)					

Note: t statistics in parentheses.

***, **, and * = p<0.01, P<0.05, P<0.1, respectively.

#### 4.3.2 Analysis of different stages of development

To evaluate the dynamic development of HSR on the consumption of urban and rural residents, we rely on the progress of “China’s railways officially entered the HSR era in 2008” and “China’s HSR exceeded 10,000 kilometers of operation in 2013, ranking first in the world”. The heterogeneity of the different stages is analyzed by dividing the study period into the following three stages: 2003–2007 for the initial stage, 2008–2012 for the steady development stage and 2013–2019 for the high-speed development stage.

In terms of CE (Table 6), the estimated coefficients for urban and rural areas from 2003 to 2007 are insignificant, while HSR from 2008 to 2012 shows a significant boost to the CE of urban and rural residents, with a greater impact on urban residents, and the coefficient for urban residents from 2013 to 2019 is insignificant, while it passes the 1% significance test for rural residents 0.027, showing that HSR further boosted the CE of rural residents. In terms of CS (Table 7), the HSR had a dampening effect and a boosting effect on the improving of the CS of urban and rural residents, respectively, only during the high-speed development phase.

**Table 6:** Regression results for different types of development (CE).

Variable	2003–2007		2008–2012		2013–2019	
	Urban	Rural	Urban	Rural	Urban	Rural
	(1)	(2)	(3)	(4)	(5)	(6)
HSR	-0.005	-0.01	0.010***	0.008*	0.004	0.027***
	(-0.77)	(-1.30)	(3.97)	(1.99)	(1.28)	(5.42)
Control	YES	YES	YES	YES	YES	YES
Constant	4.668***	1.865***	4.955***	2.108***	4.374***	0.534
	(13.03)	(3.84)	(16.52)	(5.01)	(9.13)	(0.55)
Time FE	YES	YES	YES	YES	YES	YES
City FE	YES	YES	YES	YES	YES	YES
N	150	150	150	150	210	210
R^2^	0.881	0.866	0.9	0.891	0.895	0.895

Note: t statistics in parentheses.

***, **, and * = p<0.01, P<0.05, P<0.1, respectively.

**Table 7:** Regression results for different types of development (CS).

Variable	2003–2007		2008–2012		2013–2019	
	Urban	Rural	Urban	Rural	Urban	Rural
	(1)	(2)	(3)	(4)	(5)	(6)
HSR	-0.001	0.001	0.001	0.000	-0.004**	0.002*
	(-0.90)	(0.53)	(1.16)	(-0.08)	(-2.57)	(1.86)
Control	YES	YES	YES	YES	YES	YES
Constant	0.393***	-0.011	0.179**	-0.314**	0.092	-0.520**
	(3.73)	(-0.17)	(2.47)	(-2.68)	(0.32)	(-2.75)
Time FE	YES	YES	YES	YES	YES	YES
City FE	YES	YES	YES	YES	YES	YES
N	150	150	150	150	210	210
R^2^	0.225	0.165	0.362	0.517	0.137	0.639

Note: t statistics in parentheses.

***, **, and * = p<0.01, P<0.05, P<0.1, respectively.

In summary, the effects of HSR on consumption take time to become apparent. Specifically, HSR’s contribution to rural CE increased annually and contributed significantly to the upgrading of the CS in the latter part of the period. However, HSR only boosted urban’s CE in the period 2008–2012, but hindered the upgrading of the urban CS at the end of the period.

**4.3.3 Analysis of different geographical locations.** We split the sample into eastern, central and western areas in accordance with the division of China’s three major geographical economic divisions in order to evaluate the consequence of the HSR on residents’ consumption in different geographical areas, and the results are displayed in Table 8 (only the significant results are shown). HSR boosted CE and CS in rural areas in the east, but suppressed CS in urban areas in the center and west.

**Table 8:** Regression results for different geographical locations.

	Eastern region (Rural)		Central region (Urban)	Western region (Urban)
	CE	CS	CS	CS
	(1)	(2)	(3)	(4)
HSR	0.022*	0.005**	-0.002**	-0.005**
	(2.09)	(2.63)	(-2.70)	(-3.14)
Control	YES	YES	YES	YES
Constant	1.817**	0.060	0.525***	0.282**
	(2.38)	(0.67)	(6.23)	(3.30)
Time FE	YES	YES	YES	YES
City FE	YES	YES	YES	YES
R^2^	0.94	0.574	0.479	0.375
N	204	204	153	153

Note: t statistics in parentheses.

***, **, and * = p<0.01, P<0.05, P<0.1, respectively.

Generally, rural development tends to lag behind urban development. Based on the estimation results, we conjecture that the HSR has further stimulated the consumption potential of the rural areas in the east, given that urban areas in the east are already highly developed, leaving more room for growth in rural areas. However, the HSR has contributed to the development of central areas, resulting in increased housing prices in cities along the route and creating a crowding-out effect on other forms of consumption, thereby lowering the CS in central regions. Additionally, the western region, being characterized by population outflow, has experienced a drain of young labor force due to the HSR, leaving behind a population predominantly composed of the elderly and children, who tend to prioritize survival consumption, consequently reducing the CS in western areas.

### 4.4 Mechanism test

When investigating the influence of an independent variable X on a dependent variable Y, if X affects Y indirectly through variable M, then M is termed a mediating variable. This indirect impact of X on Y through the mediating variable M is known as the mediation effect [55, 56]. The mediation model aims to uncover the causal relationships between variables, elucidating the underlying process and mechanism by which the independent variable affects the dependent variable. To validate the underlying mediating mechanisms, this section will focus on three dimensions: price effect, market effect, and income effect, to explore the mediating role of HSR in promoting the growth of CE of urban and rural residents as well as the upgrading of rural residents’ CS. Additionally, it will examine the mediating inhibitory effect of HSR on the upgrading of urban residents’ CS through the housing price effect.

#### 4.4.1 Price effect

This section selects the Consumer Price Index (CPI) for urban and rural residents as an evaluation metric for price effects, aiming to explore how HSR influences the growth of consumption expenditure of urban and rural residents and the upgrading of rural residents’ consumption structure through price effects. By thoroughly analyzing the regression results in Table 9, we can observe that in columns 2 and 7, the HSR coefficients are negative at the 1% significance level, clearly indicating that the development of HSR plays a positive role in reducing the CPI. Furthermore, the results in columns 3, 5, and 8 show that HSR effectively promotes the growth of consumption expenditure of urban and rural residents and the upgrading of rural residents’ consumption structure through price effects, with price effects playing a partial mediating role in this process.

**Table 9:** Results of price effect.

	(1)	(2)	(3)	(4)	(5)	(6)	(7)	(8)
Variables	UCE	CPI	UCE	RCE	RCE	RCS	CPI	RCS
HSR	0.007**	-0.108***	0.006**	0.019***	0.018***	0.002*	-0.090***	0.002*
	(2.56)	(-3.34)	(2.47)	(3.69)	(3.46)	(1.89)	(-3.28)	(1.71)
CPI			-0.002*		-0.008***			-0.003***
			(-1.56)		(-3.57)			(-5.30)
Control	YES	YES	YES	YES	YES	YES	YES	YES
Time FE	YES	YES	YES	YES	YES	YES	YES	YES
City FE	YES	YES	YES	YES	YES	YES	YES	YES
N	510	510	510	510	510	510	510	510
R^2^	0.970	0.144	0.970	0.963	0.963	0.731	0.161	0.741

Note: t statistics in parentheses.

***, **, and * = p<0.01, P<0.05, P<0.1, respectively.

#### 4.4.2 Market effect

Drawing upon the research of Fan et al. [57], this section selects the marketization index (MI) as a measurement of market effects. Table 10 reports the mediating effect results of HSR promoting the CE of urban and rural residents and the upgrading of rural CS through market effects. Specifically, columns 1–3 present the analysis results of urban residents’ CE, columns 2, 4, and 5 show the CE results for rural residents; and columns 6, 7, and 8 highlight the rural CS. The results indicate that both the coefficients of HSR and MI are significantly positive, suggesting that HSR can enhance the CE of urban and rural residents and the upgrading of rural CS by accelerating the process of market integration, with marketization playing a partial mediating role in this process.

**Table 10:** Results of market effect.

	(1)	(2)	(3)	(4)	(5)	(6)	(7)	(8)
Variables	UCE	MI	UCE	RCE	RCE	RCS	MI	RCS
HSR	0.007**	0.071***	0.005*	0.019***	0.016***	0.002*	0.074***	0.002*
	(2.56)	(4.37)	(1.82)	(3.69)	(2.88)	(1.89)	(4.78)	(1.57)
MI			0.028***		0.036*			0.005*
			(2.92)		(1.57)			(1.35)
Control	YES	YES	YES	YES	YES	YES	YES	YES
Time FE	YES	YES	YES	YES	YES	YES	YES	YES
City FE	YES	YES	YES	YES	YES	YES	YES	YES
N	510	510	510	510	510	510	510	510
R2	0.970	0.682	0.972	0.963	0.964	0.731	0.683	0.733

Note: t statistics in parentheses.

***, **, and * = p<0.01, P<0.05, P<0.1, respectively.

#### 4.4.3 Income effect

This section evaluates the mediating role of income effects in the promotion of urban and rural CE and the upgrading of rural CS by HSR, using the per capita disposable income (INC) of urban and rural residents as the measurement indicator. Table 11 reports the regression results for the mediating effects. Specifically, the insignificant HSR coefficients in columns 3 and 8, and the significant HSR coefficient at the 5% level in column 5, indicate that income plays a full mediating role in the enhancement of urban residents’ CE and the upgrading of rural CS driven by HSR, while exhibiting a partial mediating effect on rural CE.

**Table 11:** Results of income effect.

	(1)	(2)	(3)	(4)	(5)	(6)	(7)	(8)
Variables	UCE	INC	UCE	RCE	RCE	RCS	INC	RCS
HSR	0.007**	0.013***	0.001	0.019***	0.012**	0.002*	0.014***	0.001
	(2.56)	(4.46)	(0.40)	(3.69)	(2.52)	(1.89)	(4.30)	(1.12)
INC			0.396**		0.478***			0.071**
			(2.62)		(2.97)			(2.55)
Control	YES	YES	YES	YES	YES	YES	YES	YES
Time FE	YES	YES	YES	YES	YES	YES	YES	YES
City FE	YES	YES	YES	YES	YES	YES	YES	YES
N	510	510	510	510	510	510	510	510
R2	0.970	0.982	0.979	0.963	0.968	0.731	0.982	0.745

Note: t statistics in parentheses.

***, **, and * = p<0.01, P<0.05, P<0.1, respectively.

#### 4.4.4 Housing price effect

To verify whether the inhibiting effect of HSR on urban CS upgrading is caused by housing prices, this section further examines the mediating role of housing prices (see Table 12 for details). To distinguish from the control variable HP, this section selects the sales price of commercial housing as the proxy variable for the housing price index (HPI). The results in columns 1, 2, and 3 indicate that HSR inhibits the upgrading of urban residents’ CS by promoting an increase in housing prices, and housing prices play a partial mediating role in this process. To test the authenticity of this housing price mechanism, we further examine the rural CS. As shown in the results in column 5, the negative effect of housing prices on the upgrading of rural residents’ consumption structure is not significant.

**Table 12:** Results of housing price effect.

	(1)	(2)	(3)	(4)	(5)
Variables	UCS	HPI	UCS	RCS	RCS
HSR	-0.002*	0.009**	-0.001*	0.002*	0.003*
	(-1.76)	(2.71)	(-0.51)	(1.89)	(2.03)
HPI			-0.111*		-0.024
			(-1.87)		(-1.43)
Control	YES	YES	YES	YES	YES
Time FE	YES	YES	YES	YES	YES
City FE	YES	YES	YES	YES	YES
N	510	510	510	510	510
R^2^	0.276	0.965	0.311	0.731	0.733

Note: t statistics in parentheses.

***, **, and * = p<0.01, P<0.05, P<0.1, respectively.

## 5. Discussion and conclusions

### 5.1 Discussion

Improving transport infrastructure development is of great importance in reducing the spatial and institutional constraints that limit consumption growth. It is also a crucial step in the government’s efforts to improve people’s livelihoods and develop the country’s economy. This is the significance of the research in our article. Specifically, this study has the following significant implications. From an Academic Perspective:

First, this study reveals the significant stimulating effect of HSR on the consumption expenditure of urban and rural residents. This not only deepens our understanding of the economic effects of HSR but also provides a different perspective and empirical evidence for research in the fields of transportation economics and regional economics.

Second, the upgrading of consumption structure is an important manifestation of economic development and social progress. This study finds that the impact of HSR on urban and rural consumption is significantly heterogeneous, depending on consumption types, development stages, and geographical locations. This finding provides new academic support for understanding the differences in economic development and consumption between urban and rural areas.

From a Practical Perspective: First, the study demonstrates that HSR positively promotes the upgrading of rural residents’ consumption, while it has a certain inhibiting effect on the consumption structure upgrade of urban residents. This provides scientific evidence for the government to rationally plan the HSR network layout, optimize the allocation of urban and rural resources (especially in controlling housing prices), narrow the development gap, and promote urban-rural economic integration.

Second, the study further reveals that HSR affects the consumption of urban and rural residents through market effects, price effects, and income effects. This provides important insights for the government to formulate policies that promote the upgrading of urban and rural consumption structures. Such policies may include optimizing the consumption environment, improving consumption quality, and increasing the supply of consumer goods, effectively stimulating the consumption potential of urban and rural residents and continuously advancing the optimization and upgrading of the consumption structure.

### 5.2 Conclusions

Using panel data of urban and rural residents in 30 provinces in China from 2003 to 2019, the impact of HSR on the CE and CS of urban and rural residents in China was empirically tested through the construction of a DID model and a mediator effect model and the findings is summarized as follows:

First, HSR significantly drive the growth of urban and rural residents’ CE, particularly showcasing a notable role in promoting the upgrading of CS among rural residents, while exerting a certain inhibitory effect on the upgrading of CS among urban residents. This conclusion holds firm even after undergoing a series of robustness tests, including parallel trend tests, replacement of explanatory variables, PSM-DID method, and instrumental variable method.

Second, in the analysis of heterogeneity, we delve into the impact of HSR on different types of consumption, various stages of development, and different regions. The results indicate that the influence of HSR on urban residents’ CE primarily concentrates on housing and education-cultural entertainment items, while it demonstrates multifaceted promotion effects on rural residents’ CE, such as in education-cultural entertainment, transportation-communication, health care and medical services, clothing and housing sectors. Additionally, the impact of HSR on consumption exhibits a lag effect, notably promoting urban and rural residents’ CE after 2008 and significantly driving the upgrading of CS among rural residents after 2013 while restraining the upgrading of CS among urban residents. Simultaneously, we also uncover significant regional characteristics in the impact of HSR, notably stimulating CE and CS upgrading in eastern rural areas, yet exerting an inhibitory effect on the CS upgrading of urban residents in central and western regions.

Third, the mediation analysis unveils that HSR significantly propel the CE of urban and rural residents and upgrading of CS among rural residents through market effects, price effects and income effects. Meanwhile, HSR stimulate housing price increases, thereby restraining the upgrading of CS among urban residents.

Based on the findings of the article, the following policy recommendations are proposed: Firstly, recognizing that transportation is a pathway to prosperity, it is imperative to further enhancing the development to HSR networks, with a particular focus on rural areas. This will enable the full utilization of HSR’s stimulating effect on consumption, thereby fostering endogenous consumption growth and further driving national economic development. Secondly, acknowledging the dual nature of HSR effects, efforts should be made to mitigate negative impacts while maximizing positive outcomes. For example, local governments should proactively manage and mitigate the crowding-out impact of housing prices on the CE of local residents. Thirdly, in conjunction with improved transport infrastructure, there is necessary to further develop local specialized consumption industries, especially in rural areas. This will provide residents with a wider array of consumption choices and opportunities, thereby facilitating the upgrading of their CS.

There are certain limitations in this study. Firstly, due to the difficulty of data acquisition, the current research mainly focuses on the provincial level. In the future, if it can be extended to the prefecture-level city level for more refined analysis, it will be able to reveal more meaningful insights. Secondly, for the measurement of the explanatory variable HSR, future research can consider using more scientific and professional indicators, such as HSR network centrality and HSR accessibility, to provide more accurate assessments. Finally, given the significant spatial spillover effect of HSR, future research can further incorporate spatial factors to comprehensively and systematically analyze the spatial spillover effect of HSR on consumption.
